# Risk factors of postoperative bile leakage after liver resection: A systematic review and meta‐analysis

**DOI:** 10.1002/cam4.6128

**Published:** 2023-06-16

**Authors:** Shuai Xue, Haichuan Wang, Xiangzheng Chen, Yong Zeng

**Affiliations:** ^1^ Division of Liver Surgery, Department of General Surgery and Laboratory of Liver Surgery, and State Key Laboratory of Biotherapy and Collaborative Innovation Center of Biotherapy, West China Hospital Sichuan University Chengdu China

**Keywords:** bile leakage, liver resection, meta‐analysis, postoperative complication, risk factor

## Abstract

**Objective:**

Postoperative bile leakage (POBL) is one of the most common complications after liver resection. However, current studies on the risk factors for POBL and their impacts on surgical outcomes need to be more consistent. This study aims to conduct a meta‐analysis to analyze the risk factors for POBL after hepatectomy.

**Methods:**

We incorporated all eligible studies from Embase, PubMed, and the Web of Science database (until July 2022) into this study. RevMan and STATA software were used to analyze the extracted data.

**Results:**

A total of 39 studies, including 43,824 patients, were included in this meta‐analysis. We found that gender, partial hepatectomy, repeat of hepatectomy, extended hepatectomy, abdominal drain, diabetes, Child≥B, solitary tumor, and chemotherapy are the factors of grade B and C POBL. Some recognized risk factors were considered potential risk factors for grade B and C bile leakage because no subgroup analysis was performed, like HCC, cholangiocarcinoma, major resection, posterior sectionectomy, bi‐segmentectomy, S4 involved, S8 involved, central hepatectomy, and bile duct resection/reconstruction. Meanwhile, cirrhosis, benign diseases, left hepatectomy, and Segment 1 resection were not significant for grade B and C bile leakage. The influence of lateral sectionectomy, anterior sectionectomy, S1 involved, S3 involved, high‐risk procedure, laparoscope, and blood loss>1000 mL on POBL of ISGLS needs further research. Meanwhile, POBL significantly influenced overall survival (OS) after liver resection.

**Conclusions:**

We identified several risk factors for POBL after hepatectomy, which could prompt the clinician to decrease POBL rates and make more beneficial decisions for patients who underwent the hepatectomy.

## 1 INTRODUCTION

Liver resection is the first choice for most patients with malignant liver diseases. Hepatectomy also remains an essential treatment for patients with benign liver disease, such as liver stones, hemangiomas, and liver abscesses, in cases where medical interventions have lost their effectiveness.^1^ Due to advances in perioperative management, the incidence of post‐hepatectomy complications and mortality rates continue to decline. However, when postoperative biliary leakage (POBL) occurs, the patients undergo a painful process and even death.^2^, ^3^ Meanwhile, studies reveal that the POBL rate after hepatectomy ranged from 0.008 to 0.297 in the recent 20 years.^4^, ^5^ The latest study showed that the overall incidence of POBL is approximately 3.6%–11%.^6^ Considering the high incidence and severe impact on the postoperative course, it is crucial to understand the risk factors for POBL after hepatectomy.

Bile leakage is defined as fluid with an increased bilirubin concentration in the abdominal drain or the intra‐abdominal fluid on or after postoperative Day 3, or as the need for radiologic intervention (i.e., interventional drainage) because of biliary collections or relaparotomy resulting from bile peritonitis. Increased bilirubin concentration in the drain or intra‐abdominal fluid is defined as a bilirubin concentration at least three times greater than the serum bilirubin concentration measured simultaneously.^7^ Abdominal pain, rebound tenderness, and muscle tension is the acute symptoms. According to the international study group of liver surgery (ISGLS), POBL was classified into grades A, B, and C. Patients with grade A POBL do not need any management change in clinical practice. The remaining drainage tube shall resolve the POBL. However, patients with grade B POBL need additional diagnostic or interventional procedures without relaparotomy. Furthermore, patients with grade C POBL need relaparotomy.^8^ In the meantime, these operations could cause decreased living quality and increased operative risks. Moreover, subsequent abdominal infection or septicemia can result in longer hospital stay times, higher hospital costs, and psychological pressures for patients and their families.

The occurrence of POBL after hepatobiliary surgery may be due to the severed end of the residual bile duct, leakage at the gallbladder‐jejunostomy or T‐tube periphery, and bile duct injury due to inappropriate surgical technique.^9^ The risk factors for POBL have been extensively described recently, primarily based on univariate and multivariate analysis.^4^, ^5^, ^10^ Several studies also reported the risk factors of POBL, explicitly focusing on laparoscopic liver resection.^6^ However, these studies are inconsistent or contradictory to each other. For instance, Braunwarth et al.^11^ and Martin et al.^12^ found that major hepatectomy was always accompanied by POBL. However, Sakamoto et al.^13^ reported that minor hepatectomy, not major hepatectomy, was associated with POBL. In addition, Nakano et al.^14^ showed that BMI < 20 kg/m2 was an independent risk factor of POBL, while Cauchy et al.^15^ demonstrated that BMI > 28 kg/m^2^ is a risk factor of POBL. It is likely due to varied patient selection criteria or distinct analysis methods. Moreover, several studies said that age, sex, and diabetes were the risk factors,^7^ while most analyses reported that these factors were not. Therefore, we systemically summarized the studies on the risk factors of POBL and performed a meta‐analysis of these studies.

## 2 MATERIALS AND METHODS

This meta‐analysis followed the guidelines reported by the Preferred Reporting Items for Systematic Reviews and Meta‐Analyses (PRISMA) statement protocol.^16^ The PRISMA checklist of this meta‐analysis can be found in Table S1.

### 2.1 Search strategy

Two authors (SX and HW) independently conducted a systematic literature search. We systematically searched Embase, PubMed, and Web of Science databases until July 30, 2022. The search strategies were as follows: ((“Hepatectomy”[Mesh]) OR (liver resection)) AND (bile leakage) AND ((risk factor) OR (prognosis)) in PubMed; (((TS = (Hepatectomy)) OR TS = (liver resection)) AND TS = (bile leakage) AND TS = ((risk factor) OR (prognosis)) in Web of Science and (Hepatectomy. Mp. Or liver resection) AND (bile leakage) AND ((risk factor) OR (prognosis)) in Embase. Endnote X9 (Bld 12062, Clarivate) was applied to merge the search results and remove duplicated records according to the inclusion and exclusion criteria.

### 2.2 Inclusion and exclusion criteria

Studies were enrolled according to the inclusion and exclusion standards.

The inclusion criteria containing: (1) original English articles; (2) studies searching the variables associated with bile leakage after liver resection or the relationship between the bile leakage and prognosis; (3) randomized controlled trials (RCTs) or retrospective studies; (4) studies must include the definition of bile leakage and type of liver resection. Exclusion criteria including: (1) letters, reviews, conference abstracts, comments, case or serious reports, and experimental animal studies; (2) the duplicated cohort patients or the same data set from different studies.

## 3 DATA COLLECTION AND ANALYSIS

Two investigators selected studies and extracted data independently. When any disagreement appeared during the process, a third review author (YZ), the expert on liver surgery, resolved it by discussion. Population characteristics, intraoperative parameters, and prognosis were the primary data extracted using the standard form. Meta‐analysis was performed when the information of a parameter was available in at least four independent studies.

### 3.1 Terminologies of liver resection

The terminologies of hepatic anatomy and liver resections in the studies were mainly based on the Brisbane 2000 system,^17^, ^18^ which included extended hepatectomy, right/left (hemi‐) hepatectomy, right/left anterior/posterior/medial/lateral sectionectomy, the central hepatectomy, (bi‐/tri‐) segmentectomy, major/minor resection, and segment resection. In addition, Brisbane 2000 system excluded the non‐anatomical liver resection, so we analyzed the difference between the partial hepatectomy anatomical resection. Specifically, major hepatic resection is defined as the resection of equal to or more than three hepatic segments. In comparison, minor hepatic resection is the resection of less than three hepatic segments.^11^

### 3.2 Quality assessment

The Newcastle‐Ottawa scale (NOS) was used to assess the quality of non‐randomized studies, with a score ≥5 indicating high quality.^19^ The NOS quality scores of the studies included in our manuscript have been listed in Table S2.

### 3.3 Subgroup analysis

Because of the different definitions of POBL, a subgroup analysis was conducted to assess the potential heterogeneity between the subgroups with or without ISGLS. Meanwhile, we performed subgroup analysis with or without grade A in the studies to avoid the interference of other factors (bilioma or fluid collection) according to the ISGLS. Because grade A POBL rarely requires clinical intervention, the results could provide a reference for surgeons managing different patients. (number of studies included in each subgroup ≥2).

### 3.4 Preoperative and intraoperative risk factors

Our meta‐analysis of risk factors for POBL includes preoperative and intraoperative factors. Preoperative factors included gender, age, American Society of Anesthesiologists (ASA) classification, body mass index (BMI), cirrhosis, Child‐Pugh grade, steatosis, diabetes, hypertension, viral hepatitis, malignant disease, HCC, cholangiocarcinoma, metastatic liver tumor, colorectal metastases, benign conditions, solitary tumor, chemotherapy, portal vein embolization (PVE), and a repeat of hepatectomy.

Intraoperative factors included anatomical resection and partial hepatectomy, major resection and minor resection, extended hepatectomy, right hepatectomy, left hepatectomy, central hepatectomy, sectionectomy (lateral sectionectomy, anterior sectionectomy, posterior sectionectomy, medial sectionectomy), segmentectomy (S1‐S6‐, and S8‐ involved hepatic resection), vascular resection/anastomosis, bile duct resection/reconstruction, lymph node dissection, high‐risk procedure, laparoscope, blood loss, blood transfusions, and abdominal drain. The high‐risk procedure is defined as hepatectomy exposing the major Glissonean sheath and including the hepatic hilum on the cut surface, such as central sectionectomy, right anterior, and left medial sectionectomy and segmentectomy 5, 8.^20^, ^21^

## 4 STATISTICAL ANALYSIS

We analyzed the data using Stata (version 14.0, Stata Corporation). The prognostic value of POBL on the long‐term survival of patients who underwent the hepatectomy was estimated by the HR with 95% CI.^22^ The estimated effect measures were RR for dichotomous data and weighted mean difference (WMD) for continuous data; both were reported with 95% CI. The data heterogeneity was assessed by Cochrane's Q test (chi‐squared test) and I^2^ metric.^23^ When I^2^ > 50% indicates significant heterogeneity, the random‐effect model would be replaced with the fixed‐effect model. Publication bias was evaluated by Begg's funnel plot (qualitative) and Egger's test (quantitative). Subgroup analysis was used in Review Manager v5.3. *p* value <0.05 was considered to be statistically significant.

## 5 RESULTS

### 5.1 Evidence synthesis and characteristics of the eligible studies

**FIGURE 1:**
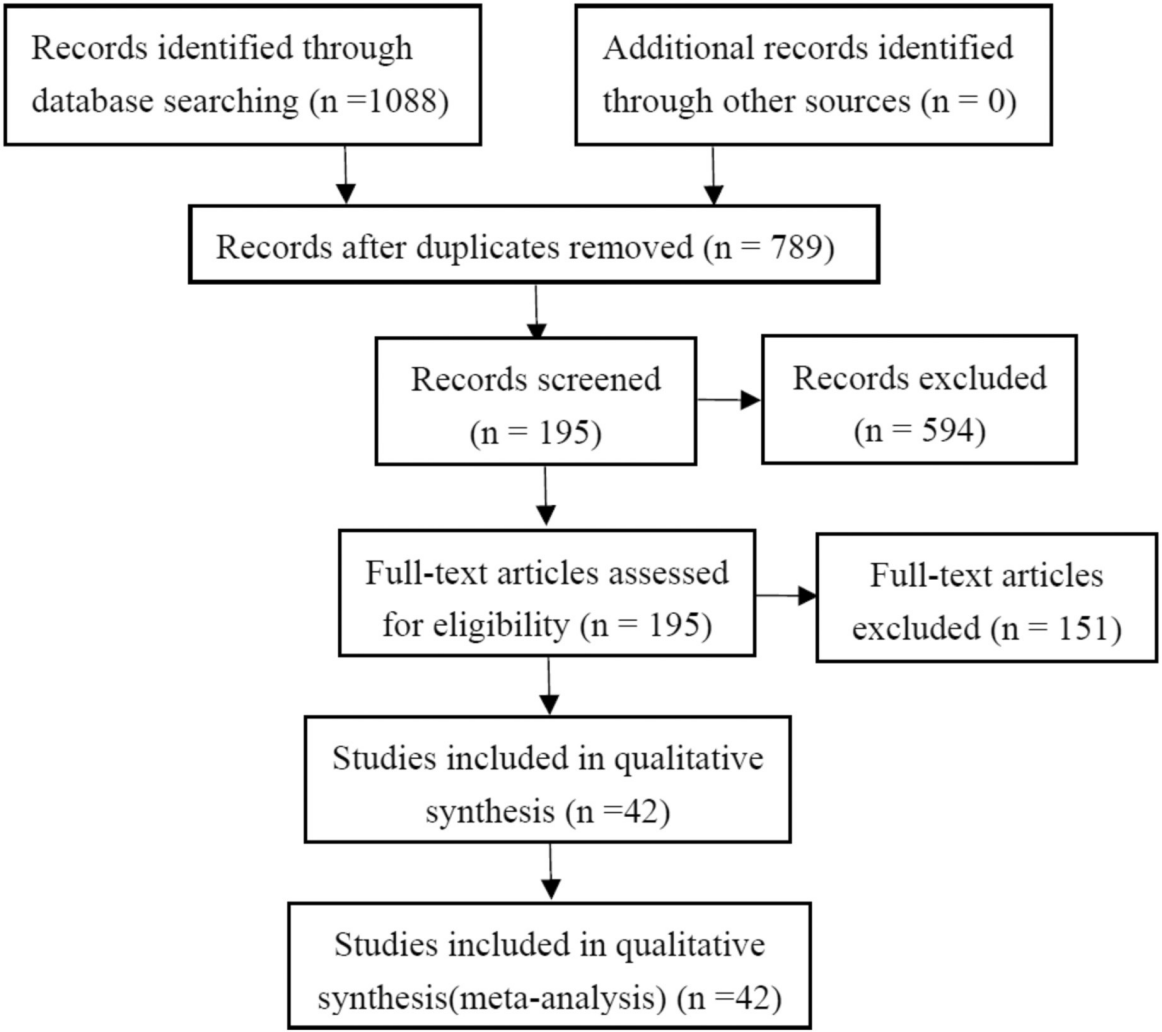
Flow diagram of the study selection process.

A total of 1088 studies were identified by using the search strategy (Figure 1). Duplicated records (*n* = 299) and records meeting the exclusion criteria (*n* = 594) were removed. The 195 papers were included for the title and abstract screening, and 42 studies with full‐text available were eventually selected for meta‐analysis. Among the included studies, 39 were used to analyze the risk factors for POBL,^4^, ^5^, ^9^, ^11^, ^12^, ^13^, ^14^, ^15^, ^20^, ^21^, ^24^, ^25^, ^26^, ^27^, ^28^, ^29^, ^30^, ^31^, ^32^, ^33^, ^34^, ^35^, ^36^, ^37^, ^38^, ^39^, ^40^, ^41^, ^42^, ^43^, ^44^, ^45^, ^46^, ^47^, ^48^, ^49^, ^50^, ^51^, ^52^ while the other 5 were used to estimate the prognostic values of POBL on liver resection.^11^, ^53^, ^54^, ^55^, ^56^

A total of 39 studies with 43,824 patients were included, with 33 (84.62%) retrospective studies and 6 (15.38%) prospective studies (Table 1). All the studies were measured with a “High” NOS quality score, indicating the comprehensive information provided (Table S2). The average/median patients' age ranged from 46.2 to 68, and the sample size ranged from 70 to 11,243. As for gender, the percentages of male patients ranged from 37% to 87%. Meanwhile, 23 studies included benign and malignant diseases, 13 investigated malignant diseases, and 3 investigated benign diseases. As to the definition of POBL, 22 studies applied the consensus from ISGLS to define POBL, and 17 studies determined POBL based on the clinical experience of the investigators. Overall, 3491 patients (8% of the included patients) experienced POBL.

**TABLE 1:** Details and the quality assessment of the included studies.

Author	Year [Ref.]	Country	Design	Age (years)	Sample size (*N*)	Male (%)	Patients with POBL (%)	Disease	NOS quality score
Yamashita et al.	2001 [24]	Japan	Retrospective	NA	781	NA	0.040	M/B	High
Tanaka et al.	2002 [20]	Japan	Retrospective	62 (3–82)	363	0.81	0.072	M	High
Nagano et al.	2003 [25]	Japan	Retrospective	62.9 (23–84)	313	0.67	0.054	M	High
Capussotti et al.	2006 [21]	Italy	Retrospective	61.7 (2–86)	610	0.60	0.036	M/B	High
Li et al.	2007 [26]	China	Retrospective	46.2 (16–76)	312	0.37	0.074	B	High
Erdogan et al.	2008 [27]	Holland	Retrospective	NA	234	0.48	0.068	M/B	High
Hayashi et al.	2010 [28]	Japan	Retrospective	NA	414	0.69	0.048	M/B	High
Hiromichi et al.	2011 [29]	Japan	Retrospective	NA	247	0.64	0.105	M/B	High
Yoshioka et al.	2011 [30]	Japan	Retrospective	64 (24–83)	505	0.63	0.067	M/B	High
Hoekstra et al.	2012 [31]	Holland	Retrospective	NA	381	0.39	0.050	M/B	High
Rahbari et al.	2012 [32]	Germany	Prospective	62 (21–88)	265	0.55	0.185	M/B	High
Sadamori et al.	2012 [33]	Japan	Retrospective	65 (32–89)	359	0.81	0.128	M	High
Guillaud et al.	2013 [10]	France	Retrospective	64 (16–90)	1001	0.68	0.080	M/B	High
Haruki et al.	2013 [34]	Japan	Retrospective	65.0 ± 10.0	105	0.73	0.086	M	High
Giuseppe et al.	2013 [35]	United States	Retrospective	57 ± 12.8	2628	0.52	0.048	M/B	High
Nanashima et al.	2013 [4]	Japan	Retrospective	NA	550	0.64	0.008	M	High
Taguchi et al.	2014 [36]	Japan	Retrospective	65.8 (22–83)	307	0.53	0.215	M/B	High
Zhang et al.	2014 [37]	China	Retrospective	49.0 ± 10.1	943	0.45	0.072	B	High
Zheng et al.	2015 [38]	China	Retrospective	57.5	297	0.43	0.226	M/B	High
Dumitrascu et al.	2016 [39]	Romania	Retrospective	59	70	0.57	0.229	M	High
Donadon et al.	2016 [40]	Italy	Retrospective	66 (23–85)	475	0.69	0.152	M	High
Kazuhiko et al.	2016 [13]	Japan	Retrospective	62 (32–87)	334	0.72	0.090	M	High
Osawa et al.	2016 [5]	Japan	Retrospective	65 (33–87)	242	0.67	0.297	M	High
Kajiwara et al.	2016 [41]	Japan	Retrospective	NA	518	0.70	0.156	M/B	High
Angela et al.	2016 [42]	South Africa	Prospective	NA	467	0.47	0.054	M/B	High
Yu et al.	2016 [43]	Seoul	Prospective	NA	154	0.66	0.188	M/B	High
Takamichi et al.	2016 [44]	Japan	Prospective	69.3	101	0.87	0.129	M	High
Bednarsch et al.	2016 [45]	Germany	Retrospective	NA	102	0.79	0.500	M	High
Dennis et al.	2016 [46]	Germany	Retrospective	59.8 ± 13.4	397	0.51	0.098	M/B	High
Francois et al.	2016 [15]	France	Retrospective	64 (24–86)	223	0.61	0.135	M/B	High
Yang et al.	2017 [47]	China	Retrospective	NA	152	0.56	0.145	B	High
Mohkam et al.	2018 [48]	France	Retrospective	63 (55–72)	778	0.58	0.082	M/B	High
Ryosuke et al.	2018 [14]	Japan	Retrospective	NA	556	0.80	0.050	M	High
Martin et al.	2018 [12]	United States	Retrospective	60 (50–68)	6859	0.48	0.077	M/B	High
Braunwarth et al.	2019 [11]	Austria	Prospective	61 (10–82)	458	0.48	0.105	M/B	High
Bagante et al.	2019 [49]	United States	Retrospective	60 (50–68)	11,243	0.51	0.078	M/B	High
Harimoto et al.	2020 [50]	Japan	Retrospective	68 (28–89)	270	0.72	0.044	M	High
Maulat et al.	2020 [51]	France	Prospective	61.5 ± 11.1	307	0.61	0.059	M/B	High
Yamashita et al.	2020 [52]	Japan	Retrospective	NA	10,102	0.80	0.072	M/B	High

*Note*: Data were expressed as mean (range) or mean ± SD. Abbreviations: B, benign disease; M, Malignant disease; NA, not available; POBL, postoperative bile leakage; Ref, reference.

### 5.2 Preoperative risk factors for POBL

We identified 24 potential preoperative risk factors for POBL that were investigated in previous studies. Studies on the specific risk factor were pooled together for analysis if the information were available. Measurements of heterogeneity were performed to determine the statistical strategies. A total of several preoperative risk factors were statistically significant, including diagnosis of cholangiocarcinoma, presence of benign liver diseases, preoperative chemotherapy, and a repeat of hepatectomy (Table 2).

**TABLE 2:** Pooled data of preoperative factors for postoperative bile leakage.

	Groups	N. studies	N. patients	BL percentage	RR (95% CI)	*p*	*p*_heterogeneity_	I^2^
Gender	Male/Female	30	23,963/15,689	0.083/0.075	1.04 (1.01–1.07)	0.004	0.471	0.0%
ASA	III IV/I II	4	1938/1734	0.066/0.061	1.11 (0.99–1.24)	0.052	0.863	0.0%
BMI	30≥/<30	4	895/2877	0.073/0.062	1.28 (0.77–2.11)	0.340	0.017	70.7%
Age	65≥/<65	6	9108/4837	0.077/0.082	1.00 (0.96–1.04)	0.829	0.378	6.0%
Cirrhosis	Yes/No	12	1807/13727	0.035/0.075	0.69 (0.57–0.82)	0.000	0.732	0.0%
Child B	B≥/<B	7	161/2753	0.087/0.086	1.26 (0.76–2.10)	0.371	0.125	39.9%
Steatosis	Yes/No	6	656/1968	0.113/0.078	1.22 (0.99–1.49)	0.058	0.985	0.0%
Diabetes	Yes/No	16	6917/28361	0.091/0.112	1.05 (0.98–1.13)	0.179	0.234	19.2%
Hypertension	Yes/No	4	6240/8587	0.075/0.074	1.04 (0.97–1.12)	0.240	0.145	44.3%
Viral hepatitis	Yes/No	9	1438/1660	0.081/0.128	0.88 (0.68–1.15)	0.362	0.001	69.3%
Malignant disease	Yes/No	7	8785/1990	0.079/0.071	1.03 (0.97–1.09)	0.334	0.001	72.9%
HCC	Yes/No	17	2801/6273	0.063/0.075	0.85 (0.76–0.95)	0.004	0.086	33.8%
Cholangiocarcinoma	Yes/No	14	596/7429	0.134/0.057	1.99 (1.60–2.46)	0.000	0.062	39.8%
Metastatic liver tumor	Yes/No	13	2285/3304	0.077/0.077	0.94 (0.84–1.05)	0.247	0.058	41.5%
Colorectal metastases	Yes/No	11	3596/3477	0.068/0.083	1.05 (0.90–1.23)	0.519	0.008	58.3%
Benign diseases	Yes/No	11	2923/14596	0.067/0.077	0.85 (0.74–0.97)	0.015	0.445	0.0%
Solitary tumors	Yes/No	7	1322/709	0.120/0.148	0.92 (0.82–1.02)	0.107	0.291	18.2%
Chemotherapy	Yes/No	11	3525/18042	0.098/0.072	1.24 (1.04–1.48)	0.014	0.022	52.1%
PVE	Yes/No	9	686/5302	0.172/0.107	1.44 (0.93–2.24)	0.105	0.000	77.9%
Arterial embolization	Yes/No	2	51/1055	0.039/0.046	0.66 (0.19–2.28)	0.508	0.930	0.0%
Previous TACE	Yes/No	2	99/483	0.152/0.174	1.18 (0.72–1.94)	0.502	0.534	0.0%
Preoperative BD	Yes/No	2	215/96	0.284/0.219	1.07 (0.94–1.21)	0.321	0.751	0.0%
Pedicle clamping	Yes/No	2	3223/8630	0.090/0.070	1.09 (0.71–1.67)	0.688	0.010	84.7%
Repeat hepatectomy	Yes/No	17	1574/13739	0.135/0.077	1.83 (1.11–3.01)	0.017	0.000	91.5%

Abbreviations: ASA, American Society of Anesthesiologists; BMI, body mass index; CI, confidence interval; HCC, hepatocellular carcinoma; PVE, portal vein embolization; RR, relative risk.

#### 5.2.1 Gender

**FIGURE 2:**
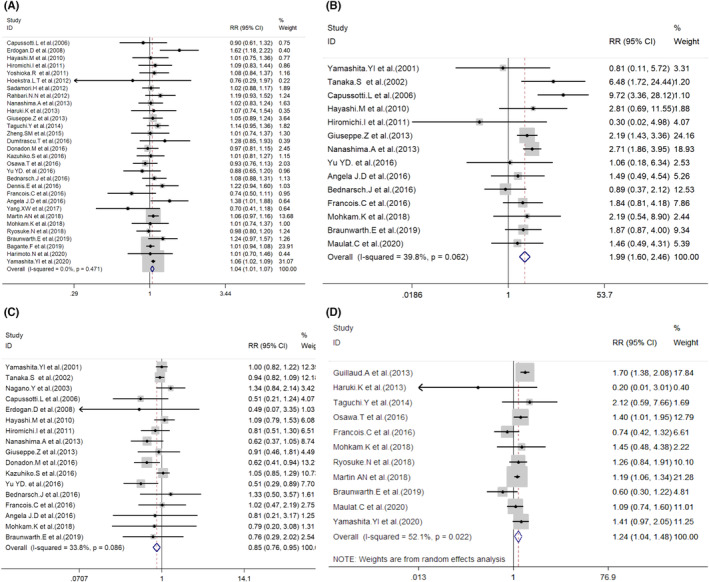
Forest plots for the association between POBL and preoperative potential risk factors. (A) Gender; (B) HCC; (C) Cholangiocarcinoma; (D) Chemotherapy. HCC, hepatocellular carcinoma.

Thirty studies^4^, ^5^, ^9^, ^11^, ^12^, ^13^, ^14^, ^15^, ^20^, ^21^, ^24^, ^25^, ^26^, ^27^, ^28^, ^29^, ^30^, ^31^, ^32^, ^33^, ^34^, ^35^, ^36^, ^37^, ^38^, ^39^, ^40^, ^41^, ^42^, ^43^, ^44^, ^45^, ^46^, ^47^, ^48^, ^49^, ^50^, ^51^, ^52^ were enrolled to investigate the impacts of gender on POBL (Table 2 and Figure 2A). For the male and female patients, the POBL rates account for 0.083 and 0.075, respectively (RR = 1.04, 95% Cl: 1.01–1.07, *p* = 0.004).

#### 5.2.2 HCC and cholangiocarcinoma

Seventeen studies^4^, ^11^, ^13^, ^15^, ^20^, ^21^, ^24^, ^25^, ^27^, ^28^, ^29^, ^35^, ^40^, ^42^, ^43^, ^45^, ^48^ focused on HCC and fourteen^4^, ^11^, ^15^, ^20^, ^21^, ^24^, ^28^, ^29^, ^35^, ^42^, ^43^, ^45^, ^48^, ^51^ studies investigated cholangiocarcinoma (Table 2). The overall POBL rates are 0.063 among patients with HCC versus 0.075 among patients without HCC (RR = 0.85, 95% Cl: 0.76–0.95, *p* = 0.004; Figure 2B). In contrast, the overall POBL rates are 0.134 among patients with cholangiocarcinoma versus 0.057 among patients without cholangiocarcinoma (RR = 1.99, 95% Cl: 1.60–2.46, *p* = 0.000; Figure 2C).

#### 5.2.3 Chemotherapy

Information on the effects of preoperative chemotherapy on POBL was retrieved from 11 studies.^5^, ^10^, ^11^, ^12^, ^14^, ^15^, ^34^, ^36^, ^48^, ^51^, ^52^ Patients who underwent chemotherapy within 1 year before hepatectomy were included for analysis. The POBL rate account for 0.098 and 0.072 in the group (RR = 1.24, 95% Cl: 1.04–1.48, *p* = 0.014) (Figure 2D).

#### 5.2.4 Repeat of hepatectomy

**FIGURE 3:**
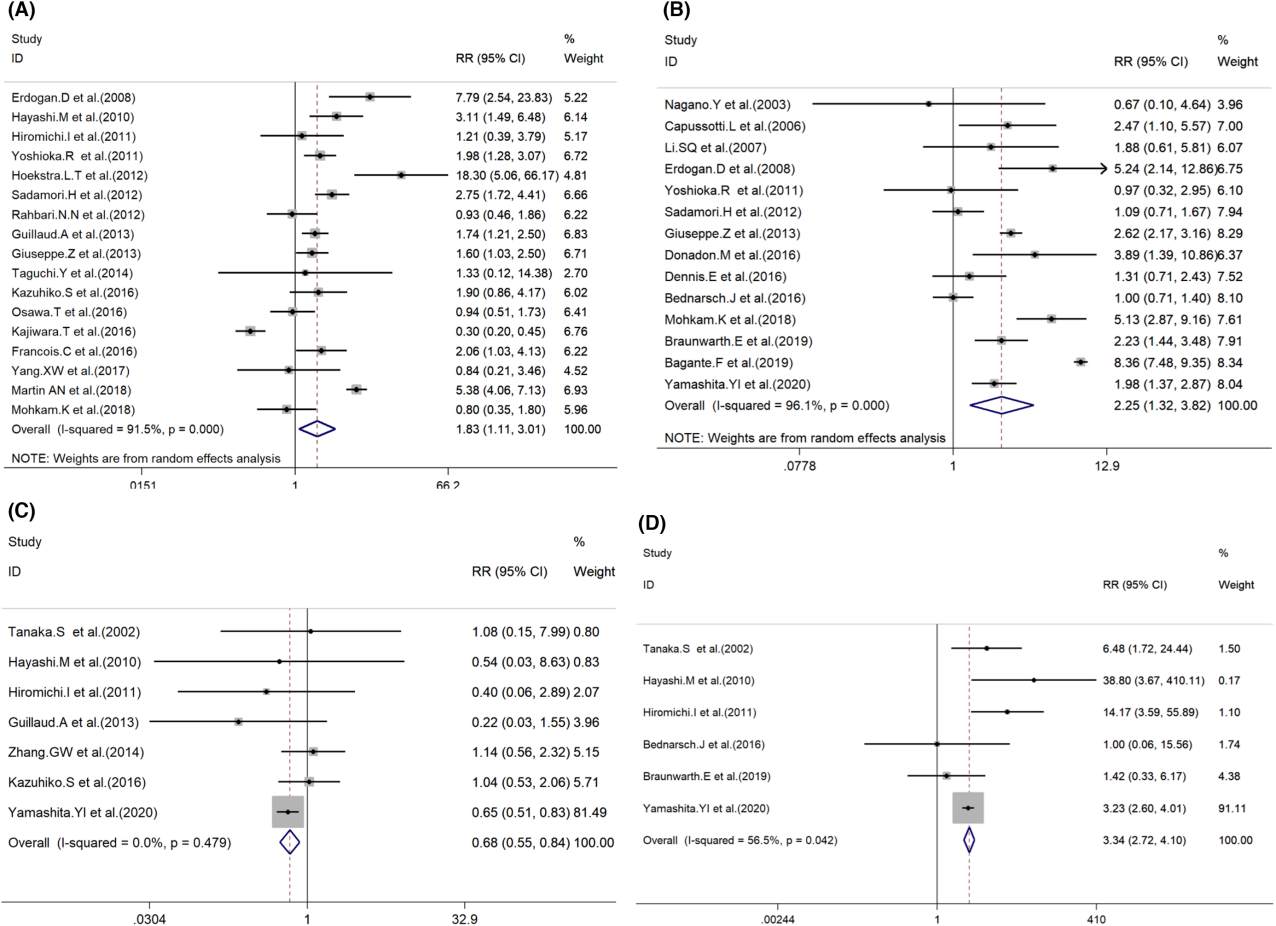
Forest plot for the association between POBL and preoperative or intraoperative potential risk factors. (A) Repeat of hepatectomy; (B) Extended hepatectomy; (C) Posterior sectionectomy; (D) Bi‐segmentectom.

There are 17 studies^5^, ^10^, ^12^, ^13^, ^15^, ^27^, ^28^, ^29^, ^30^, ^31^, ^32^, ^33^, ^35^, ^36^, ^41^, ^47^, ^48^ investigating the influence of repeat of hepatectomy on POBL. The POBL rate in the group that patients underwent repeat hepatectomy is 0.135, while the other group is 0.077 (RR = 1.83, 95% Cl: 1.11–3.01, *p* = 0.017) (Figure 3A).

### 5.3 Intraoperative risk factors for POBL

We identified 25 potential intraoperative risk factors for POBL, which were investigated in the previous studies. Studies on the specific risk factor were pooled together for analysis if the information were available. Heterogeneity measurements were performed to determine the statistical strategies, either the fixed effects model or the random effects model. Sixteen intraoperative risk factors were statistically significant, including distinct liver resection procedures, extrahepatic resections or reconstructions, and blood loss or transfusions during surgery (Tables 3 and 4).

**TABLE 3:** Pooled data of surgical procedures as the risk factors for postoperative bile leakage.

	N. studies	N. patients (Yes/No)	POBL (%) (Yes/No)	RR (95% CI)	*p*	*p*_heterogeneity_	I^2^
Hemi‐hepatectomy							
Right hepatectomy	14	3082/14260	0.065/0.076	1.07 (0.81–1.42)	0.620	0.006	55.5%
Left hepatectomy	14	3046/25373	0.099/0.073	1.14 (1.07–1.83)	0.003	0.000	78.3%
Extended hepatectomy	14	2277/26112	0.254/0.059	2.25 (1.32–3.82)	0.000	0.000	96.1%
Sectionectomy							
Lateral sectionectomy	8	562/2517	0.051/0.075	0.67 (0.49–0.90)	0.008	0.060	48.4%
Anterior sectionectomy	5	1139/10350	0.117/0.068	2.12 (1.19–3.79)	0.011	0.080	52.1%
Posterior sectionectomy	7	1516/11308	0.051/0.077	0.68 (0.55–0.84)	0.000	0.479	0.0%
Medial sectionectomy	5	567/10559	0.053/0.073	0.75 (0.52–1.06)	0.103	0.268	23.9%
Central hepatectomy	7	714/11918	0.176/0.062	3.17 (2.67–3.77)	0.000	0.000	76.5%
Bi‐segmentectomy	6	491/11189	0.212/0.071	4.72 (2.12–10.47)	0.000	0.042	56.5%
Segmentectomy							
S1 involved	14	799/7119	0.208/0.065	1.79 (1.13–2.84)	0.014	0.000	91.0%
S2 involved	4	549/1781	0.084/0.058	1.22 (0.94–1.58)	0.133	0.101	51.9%
S3 involved	5	490/2832	0.096/0.061	1.44 (1.12–1.84)	0.009	0.446	0.0%
S4 involved	5	770/1786	0.123/0.048	1.72 (1.28–2.32)	0.000	0.009	70.7%
S5 involved	5	802/2490	0.101/0.060	1.55 (0.95–2.50)	0.077	0.000	83.1%
S6 involved	4	685/2179	0.085/0.067	1.38 (0.87–2.18)	0.174	0.009	73.9%
S8 involved	6	852/2638	0.117/0.051	1.48 (1.30–1.68)	0.000	0.000	83.3%
Others							
Partial hepatectomy	16	9862/7759	0.054/0.128	0.70 (0.58–0.84)	0.000	0.001	60.3%
Anatomical resection	10	2529/2087	0.096/0.094	1.12 (0.93–1.35)	0.234	0.000	84.3%
Major Resection	16	4844/8675	0.113/0.054	1.62 (1.36–1.92)	0.000	0.000	83.1%
Minor Resection	6	953/1098	0.095/0.075	1.15 (0.85–1.55)	0.078	0.003	72.0%

Abbreviations: CI, confidence interval; RR, relative risk; S1 involved, Segment 1 involved when liver resection (other figures represent different liver segments).

**TABLE 4:** Pooled data of operation‐related factors for postoperative bile leakage.

	N. studies	N. patients (Yes/No)	POBL (%) (Yes/No)	RR (95% CI)	*p*	*p*_heterogeneity_	I^2^
Bile duct resection/reconstruction	7	619/7949	0.296/0.071	2.85 (1.68–4.82)	0.000	0.000	88.1%
Vascular resection/anastomosis	6	226/2578	0.226/0.103	2.10 (1.14–3.88)	0.017	0.015	64.4%
Lymph node dissection	5	1208/2918	0.122/0.068	1.26 (0.96–1.67)	0.101	0.000	88.2%
High‐risk procedure	6	214/2232	0.187/0.055	3.59 (1.92–6.72)	0.000	0.001	76.1%
Laparoscope	7	5249/15620	0.044/0.090	0.71 (0.43–1.17)	0.175	0.000	92.8%
Blood transfusions	16	1634/6048	0.142/0.084	1.48 (1.16–1.88)	0.002	0.000	77.1%
Blood loss >1000 mL	5	3836/10063	0.120/0.053	1.69 (1.58–1.81)	0.000	0.567	0.0%
Abdominal drain	10	5098/5505	0.122/0.042	1.41 (1.03–1.92)	0.030	0.000	98.1%

Abbreviations: CI, confidence interval; RR, relative risk.

#### 5.3.1 Hemi‐hepatectomy

A total of 42 studies mentioned the relationship between hemi‐hepatectomy and POBL, with 14 studies exploring extended right/left hepatectomy, respectively^11^, ^21^, ^25^, ^26^, ^27^, ^30^, ^33^, ^35^, ^40^, ^45^, ^46^, ^48^, ^49^, ^52^ (Table 3). Moreover, the POBL rates are 0.254 and 0.059 in the groups with or without extended hepatectomy (RR = 2.25, 95% CI: 1.32–3.82, *p* = 0.000) (Figure 3B).

#### 5.3.2 Sectionectomy

**FIGURE 4:**
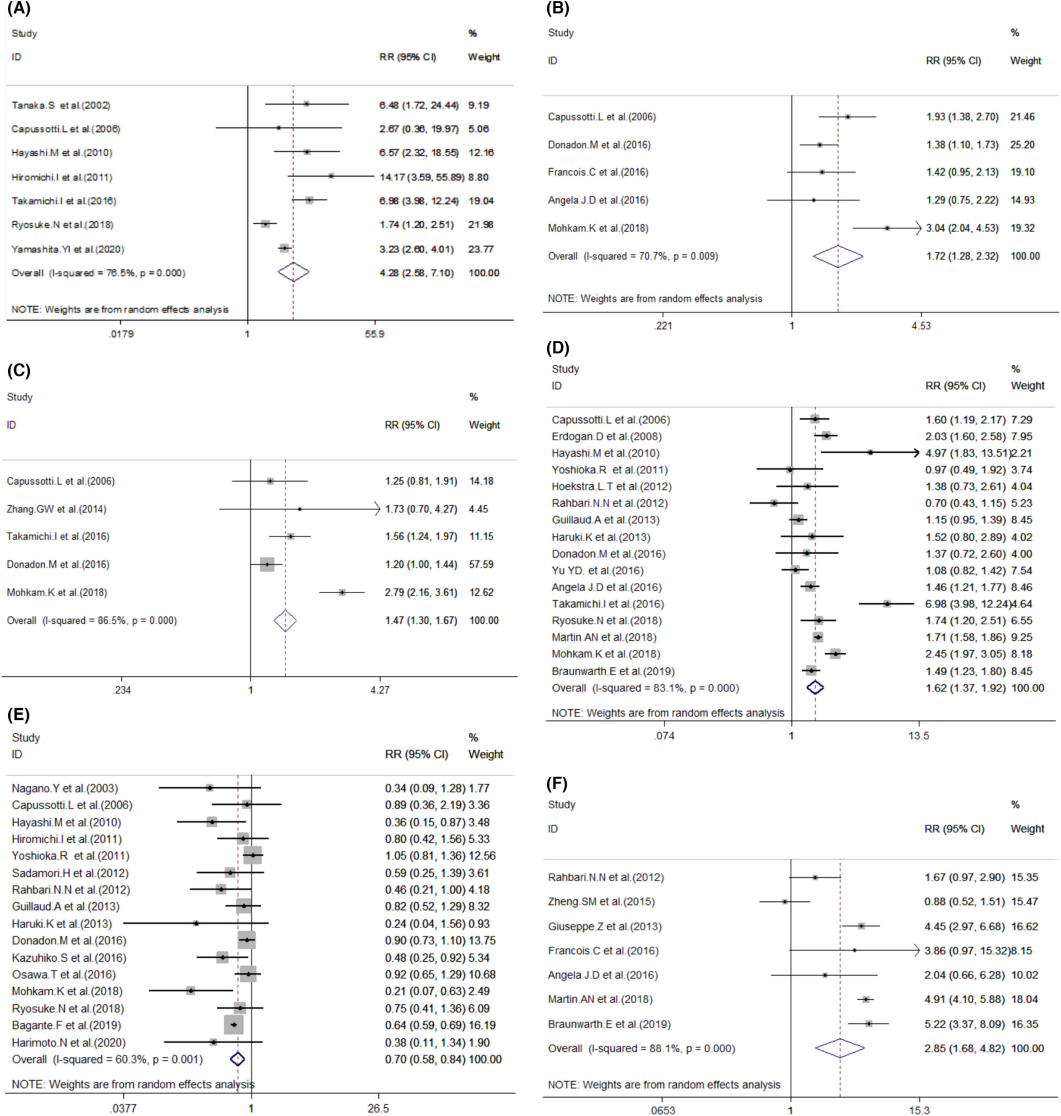
Forest plot for the association between POBL and intraoperative or other potential risk intraoperative factors. (A) Central hepatectomy; (B) S4 involved; (C) S8 involved; (D) Major resection; (E) Partial hepatectomy;(F) Bile duct resection/reconstruction.

A total of 25 studies investigated the relationship between sectionectomy and POBL, with 7 studies exploring posterior sectionectomy.^10^, ^13^, ^20^, ^28^, ^29^, ^37^, ^52^ The POBL rate for the patients with or without posterior sectionectomy is 0.051/0.077. A significant difference was observed among patients with or without posterior sectionectomy (RR = 0.68, 95% Cl: 0.55–0.84, *p* = 0.000) (Figure 3C). Of great interest, we also found that bi‐segmentectomy might also be a potential risk factor for POBL (0.212 vs. 0.071; RR = 4.72, 95% Cl: 2.12–10.47, *p* = 0.000) (Figure 3D). Moreover, the POBL rates are 0.254 and 0.059 in the groups with or without central hepatectomy^14^, ^20^, ^21^, ^28^, ^29^, ^44^, ^52^ (RR = 3.17, 95% CI: 2.67–3.77, *p* = 0.000) (Figure 4A).

#### 5.3.3 Hepatic segment resection

Several studies also investigated the effects of hepatic segmentectomy on POBL. On the one hand, some studies looked into the incidence of POBL related to the removal of specific hepatic segments; other studies analyzed the relationship between POBL rates and the amounts of the removal of hepatic segments. Specifically, hepatic resections involving in Segment 4^15^, ^35^, ^40^, ^42^, ^48^ (RR = 1.72, 95% Cl: 1.28–2.32, *p* = 0.000) (Figure 4B), and S 8^21^, ^28^, ^37^, ^40^, ^48^ (RR = 1.47, 95% Cl: 1.30–1.68, *p* = 0.000) (Figure 4C) had preliminary statistical significance. In addition, patients with and without major hepatic section,^10^, ^11^, ^12^, ^14^, ^21^, ^27^, ^28^, ^30^, ^31^, ^32^, ^34^, ^40^, ^42^, ^43^, ^44^, ^48^ which was defined as resection of equal to or more than three hepatic segments, showed a significant difference as to the rates of POBL (0.113 vs. 0.054; RR = 1.62, 95% Cl: 1.36–1.92, *p* = 0.000) (Figure 4D).

#### 5.3.4 Partial hepatectomy

Sixteen studies^5^, ^10^, ^13^, ^14^, ^21^, ^25^, ^28^, ^29^, ^30^, ^32^, ^33^, ^34^, ^40^, ^48^, ^49^, ^50^ only mentioned “partial hepatectomy” while 10 studies only analyzed the “anatomical resection” by providing more precise definitions of the surgical procedures. When analyzing this data portion, we found a significant difference in POBL rates between patients with and without partial hepatectomy (0.054 vs. 0.128; RR = 0.70, 95% Cl: 0.58–0.84, *p* = 0.000; Figure 4E).

### 5.4 Other intraoperative risk factors

**FIGURE 5:**
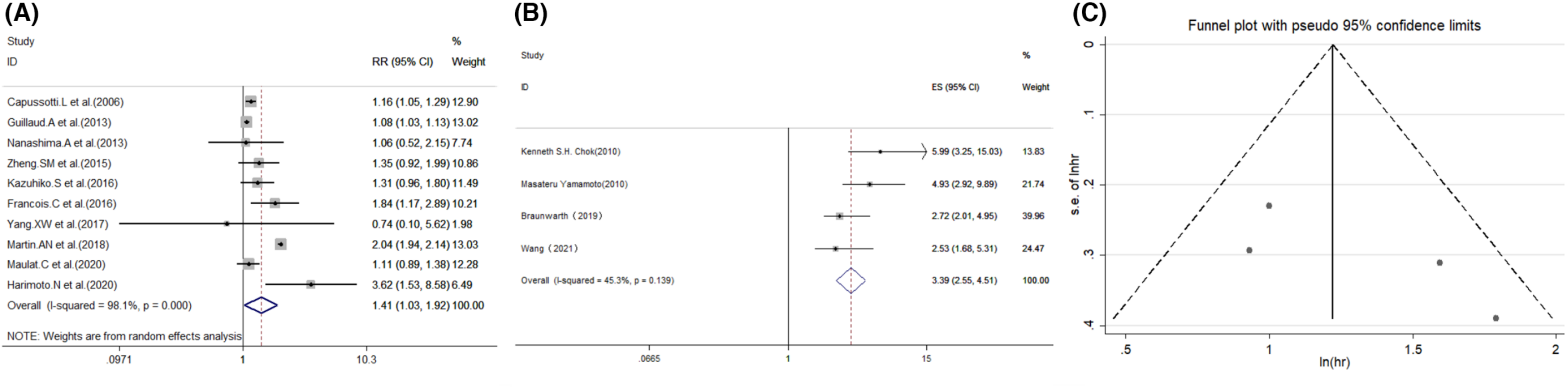
Forest plot for the association between POBL and other potential risk intraoperative factors or overall survival. (A) Abdominal drain; (B, C) overall survival and publication bias.

Next, we analyzed other potential intraoperative risk factors for POBL (Table 4). Information on bile duct reconstruction/resection about the POBL was available for seven^11^, ^12^, ^15^, ^32^, ^35^, ^38^, ^42^ studies. The results showed a significant difference between the patients with or without bile duct reconstruction or resection (0.296 vs. 0.071; RR = 2.85, 95% Cl: 1.68–4.82, *p* = 0.000) (Figure 4F). Furthermore, as the effects of abdominal drainage on POBL are inconsistent,^15^, ^50^ we then performed an analysis. The results indicated that the POBL rate of patients with abdominal drain (0.122) was also higher than those without abdominal drain^4^, ^10^, ^12^, ^13^, ^15^, ^25^, ^38^, ^47^, ^50^, ^51^ (0.042; RR = 1.41, 95% Cl: 1.03–1.92, *p* = 0.030; Figure 5A). At last, we additionally investigated whether POBL was a prognostic indicator for hepatectomy. The multivariate analysis suggested that POBL was significantly associated with poor overall survival (HR = 3.393, 95% CI: 2.553–4.509, *p* < 0.001, Figure 5B–D).

### 5.5 Subgroup analysis and multivariate analysis

In 2011, a standard concept of bile leakage was developed for clinical management and scientific research, according to the ISGLS.^7^ The definition of bile leakage has not been consistent in previous studies. In this meta‐analysis, a total of 22 studies applied the ISGLS consensus to diagnose POBL. However, 17 studies did not report the diagnostic standards or make the diagnosis based on the researchers' experience. To substantiate our analysis, we performed a subgroup analysis on those factors that were statistically different by dividing the studies into either ISGLS yes group or ISGLS no group. The results suggest that gender, benign diseases, left hepatectomy, and posterior sectionectomy have significant subgroup differences in ISGLS no group. In contrast, Segment 1 resection and blood transfusion have considerable subgroup differences in ISGLS no group (Table S3). Therefore, Segment 1 resection and blood transfusion are not the risk factor for POBL according to the ISGLS. To sum up, gender, cirrhosis, hepatocellular carcinoma, cholangiocarcinoma, benign diseases, partial hepatectomy, repeat of hepatectomy, major resection, extended hepatectomy, left hepatectomy, posterior sectionectomy, bi‐segmentectomy, S4 involved, S8 involved, central hepatectomy, bile duct resection/reconstruction seemed to be the factors for the POBL of ISGLS.

Since grade A bile leakage may be misdiagnosed due to bilioma or the fluid collection and grade A bile leakage rarely requires clinical intervention, we further performed subgroup analyses to exclude this interfering factor (Table S4). In the subgroup analysis of grade B and C bile leakage, there were significant statistical differences in liver cirrhosis, benign disease, left hepatectomy, partial hepatectomy, and blood transfusion. In the subgroup analysis of grade ABC bile leakage, there were significant statistical differences in gender. Diabetes, Child≥B, and solitary tumor initially were not statistically significant. Still, in the subgroup analysis of grade B and C bile leakage, we found that diabetes and Child≥B may be risk factors, and a single tumor may be protective factors. The current data suggest that cirrhosis, benign diseases, left hepatectomy, Segment 1 resection, and blood transfusion are not the risk factors for grade B and C POBL. The results suggest that gender, partial hepatectomy, repeat of hepatectomy, extended hepatectomy, abdominal drain, chemotherapy, diabetes, Child≥B, and solitary tumor might be the factors for POBL of grade B and C ISGLS. The remaining elements, such as liver cancer and cholangiocarcinoma, were not considered because subgroup analyses could not be performed.

## 6 DISCUSSION

Bile leakage is a common complication after hepatectomy, affecting patients' quality of postoperative life. POBL may also result in several lethal complications, including liver failure and intra‐abdominal infection.^57^ In this meta‐analysis, we systemically investigated the risk factors and the prognostic values for POBL. We identified 16 factors associated with the POBL of ISGLS. Patients with grade A POBL requires no or little change in patients' clinical management according to the ISGLS, so we conducted subgroup analyses to exclude the grade A POBL for clinicians to determine therapeutic strategies and perform perioperative care. Nine factors are regarded as the factors related to the grade B and C POBL, and we emphasized discussing these factors in the discussion.

Our study demonstrates that preoperative factors, including gender, preoperative chemotherapy, and repeat hepatectomy are factors for grade B and C POBL. This observation is based on initial and two subgroup analyses on patients, providing novel insights into the importance of gender effect on grade B and C POBL. Of great interest, gender is not considered a risk factor for grade B and C POBL occurrence previously.^26^, ^31^, ^52^ However, our meta‐analysis showed that male patients tend to have a higher risk of grade B and C POBL than female patients. One possibility might be variations in the nutritional status and body fat content between male and female patients.

Moreover, a history of chemotherapy is a risk factor for grade B and C POBL. Mechanistically, preoperative chemotherapy can cause hepatocytic atrophy and necrosis with pathologic changes and sinusoidal dilatation, resulting in slowed regeneration or healing of the remnant liver.^57^ Subgroup analysis of chemotherapy should be performed by calculating the patients of different tumor types, chemotherapy drugs, and duration of chemotherapy. Still, the included research of our study does not provide the related data. More studies must be included to investigate the relationship between chemotherapy and grade B or C POBL. In the end, consistent with previous reports, repeat hepatectomy is identified as a risk factor for grade B and C POBL, which might result from the abdominal adhesions‐induced intraoperative accidental injury.^27^, ^32^

Our study investigated the association between various liver resections and grade B and C POBL incidence according to the terminologies describing hepatectomy derived from either the Brisbane 2000 system or the Tokyo 2020 consensus. However, due to the clinical application of partial hepatectomy and the lack of data about segment resection in the included research of our studies, we also analyzed the partial hepatectomy and involved segment resection. Our results show that extended hepatectomy is the risk factor for grade B and C POBL. The studies researched by Bednarsch et al.^45^ also reported similar results, indicating the difficult recognition of the bile ducts draining of the caudate lobe or the right posterior segments that frequently drain into the left duct.^58^, ^59^ In contrast, partial hepatectomy might be the protective factor for grade B and C POBL. The reasons are likely because of the relatively small extent of partial hepatectomy compared to anatomical hepatectomy.^60^

Although the initial analysis does not regard fluid collection and bilioma, subgroup analysis suggests that the abdominal drain is still a risk factor in grade B and C POBL. An abdominal drain allows early detection of POBL, but it may also represent a risk factor for ascending and secondary infection of the collections, leading to increased severity of POBL.^61^ Worthy of mentioning, initial analysis for diabetes, Child≥ B, and solitary tumor have no statistical difference. However, in a subgroup analysis of grade B and C POBL, diabetes and Child≥ B are risk factors, whereas solitary tumor is a protective factor. This result is mainly due to the heterogeneity of included data. These results suggest that diabetes, Child≥ B, and solitary tumors are more related to the occurrence of grade B and C POBL. Diabetes, Child≥ B, and solitary tumor are not factors for grade A POBL. The relationship between three indicators and grade A POBL further analysis. Because grade B and C POBL often require clinical management, we cannot ignore these three indicators. The finding of diabetes is reasonable given that these factors may be related to liver and tissue vulnerability.^6^ Meanwhile, patients with Child B cirrhosis underwent more major hepatic resections compared to patients who did not experience biliary leakage.^62^ The resection range of multiple tumors may be more than that of the solitary tumor, so that single tumor may be a protective factor for grade B and C POBL.

Although several indicators have become recognized risk factors for POBL of ISGLS, we were not able to perform B and C POBL subgroup analyses because there were insufficient data on HCC, cholangiocarcinoma, major resection, posterior sectionectomy, bi‐segmentectomy, S4 involved, S8 involved, central hepatectomy, and bile duct resection/reconstruction. Patients diagnosed with HCC show a lower risk of POBL, whereas patients with cholangiocarcinoma tend to have a higher risk of POBL. Considering the surgeries, one crucial reason for the observed opposite readouts might be that surgeries for cholangiocarcinoma patients always involve bile duct resection and reconstruction.^63^ Several studies have shown that operations among such HCC patients were less aggressive, with a lower rate of extended and major hepatic resections.^11^ Unfortunately, the current data do not support our exploration of the effect of major and minor resection on bile leakage in patients with HCC. The same issues exist in patients with cholangiocarcinoma; no data would allow us to perform subgroup analyses of major and minor resection or bile duct resection and reconstruction. Major hepatectomy and central hepatectomy are recognized risk factors for bile leakage. Some studies have reported the risk factors of bile leakage in major hepatectomy.^15^, ^44^ We note that posterior sectionectomy might be the protective factor for POBL of ISGLS. The variations are likely because the bile ducts are generally drained into segment one and the left bile duct.^60^ However, bi‐segmentectomy seems like a risk factor for POBL of ISGLS, but which two segments need to be further studied. Central bi‐segmentectomy, S4, and S8 resection need the exposure of Glisson's sheath at the cut surface.^25^ For duct resection/reconstruction, it is an obvious risk factor for bile leakage. Although some of these indicators are recognized risk factors, we cannot definitively correlate them with grade B and grade C bile leakage because there were insufficient data to perform subgroup analyses of grade B and grade C bile leakage. Therefore, this part of the index needs to be further studied.

We explored factors insignificant in the subgroup analysis of grade B and C bile leakage but differed in the subgroup analysis of ISGLS. After we analyzed, cirrhosis, benign diseases, left hepatectomy, Segment 1 resection and blood transfusion seemed like factors for POBL of ISGLS. For example, cirrhosis patients might lead to liver parenchyma stiffness.^13^ However, Capussotti et al. found that patients with liver cirrhosis experienced a lower rate of BL during the postoperative course after hepatic resection.^21^ On the other hand, patients with cirrhosis mostly undergo minor resections. However, more studies and data are needed to clarify the effect of minor resections on postoperative bile leakage in patients with cirrhosis. Benign disease as a protective factor for POBL of ISGLS may also have a great relationship with the extent of resection. The problematic recognition of the bile ducts draining of the caudate lobe or the right posterior segments is the reason for left hepatectomy.^45^, ^58^ Segment 1 involved resection may need to expose the major Glisson's sheath and might destroy the bile duct wall.^7^ As blood transfusion is often accompanied by blood loss, it is possible that blood transfusion is an indicator of POBL. Our results show that these factors could influence the POBL of ISGLS, but grade A POBL exists in the analysis; this part of the results is only of the reference value, and there is little clinical significance.

On the other hand, the preliminary statistics of lateral sectionectomy, anterior sectionectomy, S1 involved, S3 involved, high‐risk procedure, laparoscope, and blood loss >1000 mL are significant. Because of the confounding of different POBL concepts and few studies due to subgroup analyses could not be performed, so we do not consider them. At last, bile leakage is directly related to the prognosis, which suggests that we should do an excellent clinical job of preventing and treating bile leakage and the importance of recognizing the risk factors of bile leakage, especially grade B and C POBL.

Our study provides multiple innovations in terms of surgical oncology. On the one hand, our results emphasize the importance of recognizing the risk factors of POBL among HCC patients. The latest study reports that the incidence of bile leakage after hepatectomy is about 3.6%–11%,^6^ which remains high during the decades. On the other hand, our results could prompt the clinician to decrease POBL rates and make more beneficial decisions for patients who underwent the hepatectomy, especially for patients with liver cancer. When liver cancer recurs, repeat of hepatectomy needs to be chewed over. In addition, to the best of our knowledge, we find that preoperative chemotherapy is an independent risk factor for POBL. This innovative finding hints that the administration of chemotherapy after liver resection might be an option for patients undergoing systemic therapy for advanced hepatocarcinoma. Moreover, our results as much as possible demonstrate the probability of POBL after different procedures, such as liver non‐anatomical or anatomical resection, which will provide evidence for surgeons to determine procedures for patients.

## 7 LIMITATIONS

The current meta‐analysis included results from several retrospective studies, which may affect the levels of evidence. Some studies did not use the ISGLS concept and discharge grade A bile leakage, which is the main reason for the heterogeneity of our articles. The content of previous studies has different focuses, so some missing data cannot be used to draw reliable conclusions. Further investigations with a comprehensive study design in a multicenter manner will provide reliable results.

In conclusion, the current meta‐analysis reviewed the previously reported risk factors for POBL and identified multiple preoperative or intraoperative factors that lead to a high risk of POBL. Our study will provide information for the medical team to prevent and manage POBL.

## AUTHOR CONTRIBUTIONS

**Xue Shuai:** Conceptualization (equal); writing – original draft (equal); writing – review and editing (equal). **Haichuan Wang:** Conceptualization (equal); writing – original draft (equal); writing – review and editing (equal). **Xiangzheng Chen:** Conceptualization (equal); supervision (equal); validation (equal); visualization (equal). **Yong Zeng:** Conceptualization (equal); supervision (equal); validation (equal); visualization (equal).

## FUNDING INFORMATION

This work was supported by grants from the Natural Science Foundation of China (82173124, 82173248, 82103533, 82002572, 82002967, 81972747 and 81872004).

## CONFLICT OF INTEREST STATEMENT

The authors declare that the research was conducted in the absence of any commercial or financial relationships that could be construed as a potential conflict of interest.

## Supporting information


Data S1.

## Data Availability

The raw data supporting the conclusions of this article will be made available by the authors, without undue reservation.
