# Supplementary material for: Risk factors of postoperative bile leakage after liver resection: A systematic review and meta‐analysis
Source: Cancer Med. 2023 Jun 16;12(14):14922–36. doi: 10.1002/cam4.6128 (PMC10417307; doi:10.1002/cam4.6128)
Supplement: Supplementary file 1 — Data S1. [file CAM4-12-14922-s001.pdf]

Supplementary TableS1. Systematic Reviews and Meta-Analyses (PRISMA) Statement protocol of our manuscript.

Section and Topic	Item	Checklist item	Location where item is reported
**TITLE**			
Title	1	Identify the report as a systematic review.	Page 1/Line1-2
**ABSTRACT**			
Abstract	2	See the PRISMA 2020 for Abstracts checklist.	Page 2/Line30-48/Abstract section
**INTRODUCTION**			
Rationale	3	Describe the rationale for the review in the context of existing knowledge.	Page3-4/Line50-84/Introduction, paragraph1-2
Objectives	4	Provide an explicit statement of the objective(s) or question(s) the review addresses.	Page4/Line86-101/Introduction, paragraph3
**METHODS**			
Eligibility criteria	5	Specify the inclusion and exclusion criteria for the review and how studies were grouped for the syntheses.	Page5/Line121-129/Method, paragraph3
Information sources	6	Specify all databases, registers, websites, organisations, reference lists and other sources searched or consulted to identify studies. Specify the date when each source was last searched or consulted.	Page4/Line110-119/Method, paragraph2
Search strategy	7	Present the full search strategies for all databases, registers and websites, including any filters and limits used.	Page4/Line110-119/Method, paragraph2
Selection process	8	Specify the methods used to decide whether a study met the inclusion criteria of the review, including how many reviewers screened each record and each report retrieved, whether they worked independently, and if applicable, details of automation tools used in the process.	Page4/Line104-109/Method, paragraph4
Data collection	9	Specify the methods used to collect data from reports, including how many reviewers	Page5/Line131-137/Method, paragraph4

Section and Topic	Item	Checklist item	Location where item is reported
process		collected data from each report, whether they worked independently, any processes for obtaining or confirming data from study investigators, and if applicable, details of automation tools used in the process.	
Data items	10a	List and define all outcomes for which data were sought. Specify whether all results that were compatible with each outcome domain in each study were sought (e.g. for all measures, time points, analyses), and if not, the methods used to decide which results to collect.	Page6/Line154-172/Method, paragraph7-8
	10b	List and define all other variables for which data were sought (e.g. participant and intervention characteristics, funding sources). Describe any assumptions made about any missing or unclear information.	Page6/Line154-172/Method, paragraph7-8
Study risk of bias assessment	11	Specify the methods used to assess risk of bias in the included studies, including details of the tool(s) used, how many reviewers assessed each study and whether they worked independently, and if applicable, details of automation tools used in the process.	Page6-7/Line164-174/Method, paragraph9
Effect measures	12	Specify for each outcome the effect measure(s) (e.g. risk ratio, mean difference) used in the synthesis or presentation of results.	Page6-7/Line164-174/Method, paragraph9
Synthesis methods	13a	Describe the processes used to decide which studies were eligible for each synthesis (e.g. tabulating the study intervention characteristics and comparing against the planned groups for each synthesis (item #5)).	Page6-7/Line154-174/Method, paragraph7-9
	13b	Describe any methods required to prepare the data for presentation or synthesis, such as handling of missing summary statistics, or data conversions.	Page6-7/Line154-174/Method, paragraph7-9
	13c	Describe any methods used to tabulate or visually display results of individual studies and syntheses.	Page6-7/Line154-174/Method, paragraph7-9

Section and Topic	Item	Checklist item	Location where item is reported
	13d	Describe any methods used to synthesize results and provide a rationale for the choice(s). If meta-analysis was performed, describe the model(s), method(s) to identify the presence and extent of statistical heterogeneity, and software package(s) used.	Page6-7/Line164-190/Method, paragraph9-10
	13e	Describe any methods used to explore possible causes of heterogeneity among study results (e.g. subgroup analysis, meta-regression).	Page6-7/Line164-190/Method, paragraph9-10
	13f	Describe any sensitivity analyses conducted to assess robustness of the synthesized results.	Page6-7/Line164-190/Method, paragraph9-10
Reporting bias assessment	14	Describe any methods used to assess risk of bias due to missing results in a synthesis (arising from reporting biases).	Page6-7/Line164-190/Method, paragraph9-10
Certainty assessment	15	Describe any methods used to assess certainty (or confidence) in the body of evidence for an outcome.	Page6-7/Line164-190/Method, paragraph9-10
**RESULTS**			
Study selection	16a	Describe the results of the search and selection process, from the number of records identified in the search to the number of studies included in the review, ideally using a flow diagram.	Page7/Line194-200/result, paragraph1
	16b	Cite studies that might appear to meet the inclusion criteria, but which were excluded, and explain why they were excluded.	Page7/Line194-200/result, paragraph1
Study characteristics	17	Cite each included study and present its characteristics.	Page7-8/Line194-212/result, paragraph1-2
Risk of bias in studies	18	Present assessments of risk of bias for each included study.	Page8/Line202-212/result, paragraph2/table1
Results of individual	19	For all outcomes, present, for each study: (a) summary statistics for each group (where	Page8/Line202-212/result, paragraph2

Section and Topic	Item	Checklist item	Location where item is reported
studies		appropriate) and (b) an effect estimate and its precision (e.g. confidence/credible interval), ideally using structured tables or plots.	
Results of syntheses	20a	For each synthesis, briefly summarise the characteristics and risk of bias among contributing studies.	Page8-13/Line214-356/result, paragraph3-14/table2-4/figure1-4/supplementary
	20b	Present results of all statistical syntheses conducted. If meta-analysis was done, present for each the summary estimate and its precision (e.g. confidence/credible interval) and measures of statistical heterogeneity. If comparing groups, describe the direction of the effect.	Page8-13/Line214-356/result, paragraph3-14/table2-4/figure1-4/supplementary
	20c	Present results of all investigations of possible causes of heterogeneity among study results.	Page8-13/Line214-356/result, paragraph3-14/table2-4/figure1-4/supplementary
	20d	Present results of all sensitivity analyses conducted to assess the robustness of the synthesized results.	Page8-13/Line214-356/result, paragraph3-14/table2-4/figure1-4/supplementary
Reporting biases	21	Present assessments of risk of bias due to missing results (arising from reporting biases) for each synthesis assessed.	Page8-13/Line214-356/result, paragraph3-14/table2-4/figure1-4/supplementary
Certainty of evidence	22	Present assessments of certainty (or confidence) in the body of evidence for each outcome assessed.	Page8-13/Line214-356/result, paragraph3-14/table2-4/figure1-4/supplementary
**DISCUSSION**			
Discussion	23a	Provide a general interpretation of the results in the context of other evidence.	Page13-17/Line358-473/discussion, paragraph1-8
	23b	Discuss any limitations of the evidence included in the review.	Page13-17/Line358-473/discussion, paragraph1-8
	23c	Discuss any limitations of the review processes used.	Page13-17/Line358-473/discussion,

Section and Topic	Item	Checklist item	Location where item is reported
			paragraph1-8
	23d	Discuss implications of the results for practice, policy, and future research.	Page17/Line470-473/discussion, paragraph8
**OTHER INFORMATION**			
Registration and protocol	24a	Provide registration information for the review, including register name and registration number, or state that the review was not registered.	N/A
	24b	Indicate where the review protocol can be accessed, or state that a protocol was not prepared.	N/A
	24c	Describe and explain any amendments to information provided at registration or in the protocol.	N/A
Support	25	Describe sources of financial or non-financial support for the review, and the role of the funders or sponsors in the review.	Page17/Line479-481/ Funding section
Competing interests	26	Declare any competing interests of review authors.	Page17/Line483-486/ Conflict of interest section
Availability of data, code and other materials	27	Report which of the following are publicly available and where they can be found: template data collection forms; data extracted from included studies; data used for all analyses; analytic code; any other materials used in the review.	N/A

N/A: Not Applicable.

Supplementary TableS2. The NOS quality score of the articles included in this study.

Author	Case Control Studies/ Cohort Studies							NOS Quality Score
	Selection			Comparability			Exposure/Outcome	
Yamashita.YI et al.	*	*	*		*	*		5
Tanaka.S et al.	*	*	*		*	*		5
Nagano.Y et al.	*	*	*		*	*		5
Capussotti.L et al.	*	*	*		*	*	**	7
Li.SQ et al.	*	*	*		*	*		5
Erdogan.D et al.	*	*	*		*	*	*	6
Hayashi.M et al.	*	*	*		*	*	*	6
Hiromichi.I et al.	*	*	*		*	*	*	6
Yoshioka.R et al.	*	*	*		*	*	*	6
Guillaud.A et al.	*	*	*		*	*		5
Hoekstra.L.T et al.	*	*	*		*	*		5
Rahbari.N.N et al.	*	*	*		*	*	**	7
Sadamori.H et al.	*	*	*		*	*	*	6
Haruki.K et al.	*	*	*		*	*	**	7
Giuseppe.Z et al.	*	*	*		*	*	*	6
Nanashima.A et al.	*	*	*		*	*	*	6
Taguchi.Y et al.	*	*	*		*	*	**	7
Zhang.GW et al.	*	*	*		*	*	*	6
Dumitrascu.T et al.	*	*	*		*	*		5
Donadon.M et al.	*	*	*		*	*	*	6
Kazuhiko.S et al.	*	*	*		*	*	*	6
Osawa.T et al.	*	*	*		*	*	**	7

Kajiwara.T et al.	*	*	*		*	*	*	6
Angela J.D et al.	*	*	*		*	*	*	6
Yu YD. et al.	*	*	*		*	*	**	7
Takamichi.I et al.	*	*	*		*	*	*	6
Bednarsch.J et al.	*	*	*		*	*	**	7
Dennis.E et al.	*	*	*		*	*	**	7
Francois.C et al.	*	*	*		*	*	**	7
Zheng.SM et al.	*	*	*		*	*	*	6
Yang.XW et al.	*	*	*		*	*	**	7
Mohkam.K et al.	*	*	*		*	*	*	6
Ryosuke.N et al.	*	*	*		*	*	*	6
Maulat.C et al.	*	*	*		*	*		5
Martin.AN et al.	*	*	*		*	*	*	6
Braunwarth.E et al.	*	*	*		*	*		5
Bagante.F et al.	*	*	*		*	*	*	6
Harimoto.N et al.	*	*	*		*	*		5
Yamashita.YI et al.	*	*	*		*	*	*	6

Abbreviations: NOS:Newcastle-Ottawa Scale.

Supplementary Table S3. Subgroup analysis of different definition of POBL.

	ISGLS	N. studies	OR(95%CI)	Subtotal of subgroups		
				P	$P_{heterogeneity}$	I^2
Gender	Yes	18	1.11(1.01-1.20)	0.02	0.28	15%
	No	12	1.19(0.97-1.46)	0.09	0.74	0%
Cirrhosis	Yes	6	0.65(0.51-0.83)	0.004	0.74	0.0%
	No	6	0.62(0.40-0.95)	0.03	0.32	15%
Hepatocellular carcinoma	Yes	7	0.64(0.45-0.92)	0.02	0.34	12%
	No	10	0.67(0.51-0.88)	0.004	0.26	20%
Cholangiocarcinoma	Yes	7	2.45 (1.68-3.58)	0.000	0.62	0%
	No	7	2.04 (1.39-3.00)	0.0003	0.01	63%
Benign diseases	Yes	6	0.82(0.69-0.96)	0.02	0.13	41%
	No	5	0.83(0.47-1.47)	0.52	0.83	0.0%
Partial hepatectomy	Yes	8	0.67(0.54-0.82)	0.000	0.006	65%
	No	8	0.73(0.54-0.99)	0.005	0.10	42%
Repeat of hepatectomy	Yes	10	1.43(1.18-1.73)	0.0003	0.000	95%
	No	7	2.00(1.46-2.75)	0.0000	0.001	72%
Major Resection	Yes	8	1.61(1.52-1.72)	0.000	0.00	89%
	No	8	1.56(1.48-1.66)	0.007	0.00	76%
Segment 1 resection	Yes	8	1.29(0.90-1.86)	0.16	0.14	36%
	No	6	2.74(1.95-3.86)	0.000	0.37	8%
Extended hepatectomy	Yes	5	5.34(4.85-5.89)	0.000	0.000	98%
	No	9	2.01(1.73-2.33)	0.000	0.000	82%
Left hepatectomy	Yes	5	1.22(1.04-1.42)	0.01	0.000	84%
	No	9	1.28(0.92-1.77)	0.14	0.01	60%

Posterior sectionectomy	Yes	3	0.62(0.48-0.80)	0.0002	0.28	21%
	No	4	0.89(0.44-1.82)	0.76	0.75	0.0%
Bisegmentectomy	Yes	2	3.15(2.54-3.90)	0.000	0.278	15.1%
	No	4	7.46(3.50-15.91)	0.000	0.186	37.7%
S4 involved	Yes	3	2.37(1.52-3.71)	0.0002	0.06	64%
	No	2	2.23(1.44-3.46)	0.0003	0.24	29%
S8 involved	Yes	2	7.38(3.47-15.72)	0.000	0.89	0%
	No	3	1.62(1.07-2.45)	0.02	0.96	0%
Central hepatectomy	Yes	3	3.59(2.86-4.52)	0.000	0.04	69%
	No	4	8.17(4.02-16.61)	0.000	0.57	53%
Bile duct resection/reconstruction	Yes	5	3.73(3.20-4.34)	0.000	0.000	90.0%
	No	2	2.91(2.11-4.02)	0.000	0.004	87.7%
Blood transfusion	Yes	9	1.12(0.95-1.32)	0.192	0.000	78%
	No	7	1.92(1.65-2.22)	0.000	0.159	35%

Abbreviations: ISGLS: the international study group of liver surgery; POBL: post-operative bile leakage; OR: odds ration; CI: Confidence interval.

Supplementary Table S4. Subgroup analysis of different definition of grade B and C POBL.

	Grade	N. studies	OR(95%CI)	Subtotal of subgroups		
				P	$P_{heterogeneity}$	I^2
Gender	BC	8	1.29(1.09-1.52)	0.003	0.90	0.0%
	ABC	10	1.04(0.94-1.15)	0.41	0.18	29%
Cirrhosis	BC	2	0.49(0.17-1.42)	0.19	0.74	0%
	ABC	3	0.43(0.21-0.85)	0.02	0.76	0%
Benign diseases	BC	2	0.90(0.36-2.24)	0.82	0.05	73%
	ABC	4	0.76(0.64-0.90)	0.002	0.06	60%
Partial hepatectomy	BC	4	0.17(0.10-0.29)	0.000	0.000	87%
	ABC	4	0.43(0.28-0.65)	0.000	0.000	93%
Repeat of hepatectomy	BC	3	1.94 (1.16-3.25)	0.01	0.03	73%
	ABC	6	1.36(1.11-1.68)	0.004	0.000	97%
Extended hepatectomy	BC	2	1.84(1.29-2.62)	0.000	0.28	16%
	ABC	3	37.82(30.48-46.92)	0.000	0.000	96%
Left hepatectomy	BC	3	0.94(0.75-1.18)	0.53	0.000	86%
	ABC	2	1.55 (1.26-1.90)	0.000	0.16	49%
Segment 1 resection	BC	5	1.14(0.73, 1.78)	0.57	0.49	0%
	ABC	3	1.70 (0.92, 3.14)	0.09	0.08	61%
Blood transfusion	BC	4	0.93(0.65-1.33)	0.70	0.000	87%
	ABC	4	1.79(1.23-2.62)	0.002	0.000	56%
Abdominal drain	BC	2	1.80(1.06-3.06)	0.03	0.10	64%
	ABC	7	5.44(4.47-6.62)	0.000	0.000	84%
Diabetes	BC	5	1.24 (1.08-1.43)	0.03	0.90	0%

	ABC	8	0.92(0.80-1.05)	0.21	0.30	16%
Child⩾B	BC	3	2.20 (1.11-4.38)	0.02	0.22	33%
	ABC	2	1.17 (0.34-4.00)	0.80	0.75	0%
Solitary tumor	BC	2	0.58(0.36-0.93)	0.03	0.70	0%
	ABC	3	1.12(0.74-1.70)	0.60	0.64	0%
Chemotherapy	BC	4	1.48 (1.07-2.05)	0.02	0.95	0%
	ABC	6	1.35 (1.15-1.57)	0.000	0.004	71%

Abbreviations: ISGLS: the international study group of liver surgery; POBL: post-operative bile leakage; OR: odds ration; CI: Confidence interval.
